# Editorial: Cognition, neurodegeneration and immunity: from observational data to molecular mechanisms

**DOI:** 10.3389/fnhum.2025.1704828

**Published:** 2025-10-20

**Authors:** George D. Vavougios, Paul Edison, Giovanni D'Avossa

**Affiliations:** ^1^Medical School, University of Cyprus, Nicosia, Cyprus; ^2^Department of Brain Sciences, Faculty of Medicine, Imperial College London, London, United Kingdom; ^3^School of Psychology and Sports Sciences, Bangor University, Bangor, United Kingdom

**Keywords:** cognition, infection–immunology, neurodegeneration, immunity, neuroscience

The enduring interconnection between immunology, cognitive aging, and neurodegeneration has become a defining axis in contemporary neuroscience. The consequences of infection and dysregulated immunity have often provided an opportunity to study this tripartite relationship, as well as individual connections between its constituents.

Perhaps even more so amid the COVID-19 pandemic, the neurological and cognitive repercussions that followed infection elevated urgent questions about the immune system's role in cognitive decline and long-term brain health.

These sources of inspiration lead us to develop *Cognition, Neurodegeneration and Immunity: From Observational Data to Molecular Mechanisms*, a Research Topic that embraces an integrative approach—from observational clinical data to mechanistic modeling—seeking convergence across epidemiology, immunology, and translational neuroscience. Several relevant works have since elucidated this timely research question, and are part of this overarching theme.

## Clinical observations illuminate age-dependent outcomes

The emergence of Long COVID in the aftermath of the forced clinicians and researchers to reconsider the spectrum of post-viral illnesses. The potential of long-lasting impairment of cognitive functions was captured in Gonzalez Aleman et al.'s landmark study, “*Age-dependent phenotypes of cognitive impairment as sequelae of SARS-CoV-2 infection*,” reporting on data from a multinational cohort. By stratifying cognitive impairments based on age, the authors highlight that older adults exhibit disproportionately severe and persistent deficits following exposure to COVID-19—underscoring vulnerability nestled in immunosenescence and neuroinflammatory priming in aging. This work not only contributes to clinical datasets by a unique cohort, but also serves as a foundation for mechanistic hypotheses, emphasizing the urgency of age-sensitive intervention strategies.

## Immunity and vulnerability in special populations

A similarly compelling dimension of the pandemic and its impact on the nervous system emerges in transplanted patients. The study on *Neurological complications of SARS-CoV-2 infection among solid-organ transplanted patients: does immunosuppression matter?* by Avorio et al. examines whether immunosuppressive regimens modulate the risk or character of neurological sequelae. Surprisingly, the findings suggest that while immunosuppression might mask certain inflammation-related symptoms, it also opens a window into critical immune thresholds that may result in CNS involvement. These insights hint at a nonlinear role of immune activation, suggesting both damaging over-activation and protective dampening must be considered.

## Case report: peripheral autoimmunity, central implications

Autoimmune nodopathy is a rare demyelinating polyneuropathy driven by IgG4-subclass autoantibodies targeting peripheral nodal and paranodal proteins, with anti-neurofascin-155 (NF155) being the most common autoantibody identified (Sharma et al., 2024).

Notably, neurofascin antibodies have also been identified in patients with central demyelinating disease including multiple sclerosis and combined central and peripheral demyelination (CCPD). Although autoimmune nodopathies have received increased recognition in recent literature, their phenotypic spectrum may still be expanding—particularly regarding the involvement of the central nervous system.

In this context, the case report *Therapy-resistant autoimmune nodopathy with anti-neurofascin 155 antibodies* (Talers et al.). underscores how autoimmunity against cell adhesion molecules can precipitate profound neurological symptoms, as well as the importance of incorporating knowledge of immune—neuronal interactions in complex treatment decisions.

## Mechanistic clarifications: interferon's double edge

Cao's mini-review, *In sickness and in health—Type I interferon and the brain*, distills decades of evidence on type I interferon tonicity and its oscillation between protective antiviral roles and neurotoxic consequences in the setting of chronic activation. The article expands on molecular cascades whereby sustained interferon activity disrupts synaptic homeostasis, impairs neurogenesis, and potentiates microglial-mediated synaptic pruning—mechanisms with direct relevance to cognitive impairment. This thematic anchor offers indispensable context for understanding the pathophysiology emerging from observational studies.

The accompanying Perspective by Vavougios et al., *Type I interferon signaling, cognition and neurodegeneration following COVID-19*, advances a sophisticated model interweaving immune activation with Alzheimer's-related processes. The authors propose that persistent interferon signaling—triggered by systemic infection—can intersect with amyloid and tau pathology, effectively bridging infectious disease models with classical neurodegenerative frameworks. It is a foundational viewpoint for translating COVID-19–related neuroimmune changes toward broader neurodegeneration pathobiology.

## Integrating across observational and mechanistic domains

This Research Topic's focal point was the investigation of neuroimmune dynamics, integrating multi-level data: from clinical datasets to molecular mechanisms. Our topic focuses on human epidemiology as the premise for age-specific cognitive sequelae, seeking to identify clinical vulnerabilities like immunocompromised states. Molecular insights into interferon-driven neurotoxicity serve to identify how immune pathways can plausibly link clinical observations onto established neurodegenerative models. The juxtaposition of clinical observation and immunological mechanics offers a multidimensional view of cognition as within the constraints of immune functions and the concept of innate immune tonicity as a continuum between health and disease.

## Conclusion

We extend our gratitude to the contributors for strategic, interdisciplinary scholarship that spans clinical cohorts, case pathology, and molecular models. This body of work advances our understanding of how immune signals—from systemic infection to localized inflammation—can sculpt cognitive outcomes and potentially accelerate neurodegeneration. It is our hope that this Research Topic not only guides future research but also catalyzes cross-disciplinary collaborations that will translate into immune-informed diagnostics and therapies.
